# Endovascular repair of traumatic axillosubclavian artery injuries

**DOI:** 10.1016/j.jvscit.2021.11.006

**Published:** 2021-12-08

**Authors:** Jason Zhang, Rohan Basu, Andrew R. Bauder, Jon G. Quatramoni, Julia Glaser, Venkat Kalapatapu, Ann C. Gaffey

**Affiliations:** a Division of Vascular and Endovascular Surgery, Department of Surgery, Perelman School of Medicine, University of Pennsylvania, Philadelphia, Pa; b Division of Plastic and Reconstructive Surgery, Department of Surgery, Perelman School of Medicine, University of Pennsylvania, Philadelphia, Pa; c Department of Vascular Surgery, Cleveland Clinic, Cleveland, Ohio; d Division of Vascular and Endovascular Surgery, Department of Surgery, University of California, San Diego, School of Medicine, La Jolla, Calif

**Keywords:** Axillary artery, Subclavian artery, Trauma

## Abstract

Gun violence reached a 20-year peak in 2020, with the first-line treatment of axillosubclavian vascular injuries (SAVIs) remaining unknown. Traditional open exposure is difficult and exposes patients to iatrogenic venous and brachial plexus injury. The practice of endovascular treatment has been increasing. We performed a retrospective analysis of SAVIs at a level I trauma center. Seven patients were identified. Endovascular repair was performed in five patients. Technical success was 100%. The early results suggest that endovascular treatment of trauma-related SAVIs can be performed safely and effectively. However, complications such as stent thrombosis or occlusion can occur, demonstrating the need for surveillance.

In 2020, firearm violence was responsible for nearly 20,000 homicides in the United States, reaching a 20-year peak.^1^ The incidence of axillosubclavian arterial injuries (SAVIs) has been low owing to the surrounding bony structures and the high pre- and in-hospital mortality secondary to rapid exsanguination and associated injuries.^2^, ^3^, ^4^ SAVIs can be especially difficult to treat owing to the complex regional anatomy, complicated exposure, presence of associated injuries, and limited clinical experience.

The surgical management of penetrating SAVIs has varied. Low-grade injuries, including minimal intimal injuries or occlusion without evidence of limb ischemia, can be managed nonoperatively with observation, serial imaging studies, and antiplatelet therapy.^3^ Higher grade injuries, including disruption or occlusion with evidence of ischemia, have traditionally involved an open surgical approach entailing a clavicular incision and median sternotomy or anterolateral thoracotomy for adequate exposure and control.^2^^,^^5^ Such open interventions have had a high rate of morbidity.^6^, ^7^, ^8^

Endovascular surgical options have continued to increase in popularity for the management of SAVIs in appropriate patients.^9^^,^^10^ Although mostly studied in hemodynamically stable patients, endovascular management of SAVIs has been associated with shorter operative times and less blood loss compared with open approaches with noninferior outcomes.^11^, ^12^, ^13^ Thus, we reviewed our contemporary experience of SAVIs treated endovascularly.

## Methods

After approval from our institution's institutional review board, a retrospective medical record review was performed of a prospectively maintained trauma database. All patients who had presented with subclavian, axillary, brachial, and/or innominate artery injuries to the level 1 trauma center between January 2015 and January 2020 were reviewed. The outcome measures included technical success, hospital mortality, operative time, early (<30 days) complications, late (>30 days) complications, and reintervention rates.

## Results

### Initial treatment

During the study period, seven patients were identified who had sustained a SAVI. Six of the patients were men, with a mean age of 29.3 years (range, 21-41 years). Six injuries had resulted from penetrating trauma. Two patients had presented in cardiogenic and/or hypovolemic shock and one had undergone unsuccessful thoracotomy in the emergency department. The injury specifics are listed in Table I. A massive transfusion protocol was initiated for both hemodynamically unstable patients. The vascular pathology found on the imaging studies included two complete transections, one occlusion, one laceration, one pseudoaneurysm, and two cases thought to be vasospasm. Definitive signs of vascular trauma included one patient with active pulsatile bleeding and five patients with a loss of the radial pulse. The mean injury severity score for all seven patients was 29.6 (range, 1-75). The demographic and clinical features are listed in Table II.

**Table I:** Injury characteristics of study patients (n = 7)

Variable	No. (%)
Artery injured	
Axillary	2 (28)
Subclavian	3 (43)
Innominate	1 (14)
Brachial	1 (14)
Injury	
Transection	2 (28)
Laceration	1 (14)
Pseudoaneurysm	1 (14)
Vasospasm	2 (28)
Occlusion	1 (14)

**Table II:** Demographics of patients who had undergone initial endovascular therapy (n = 5)

Variable	Mean (range) or no. (%)
Age, years	28 (21-38)
Male sex	5 (100)
Injury distribution	
Blunt	1 (20)
Penetrating	4 (80)
Mechanism	
Gunshot wound	3 (60)
Stabbing	1 (20)
Motor vehicle collision	1 (20)
Presenting definitive signs	
Hypotension (SBP <90 mm Hg)	2 (40)
Motor/sensory deficit	2 (40)
Active pulsatile bleeding	1 (20)
Diminished pulses	4 (80)
Injury severity score	26.2 (15-41)
Patients with preoperative CTA	5 (100)
Stents	
Self-expanding Viabahn stent	4 (80)
Balloon-expandable VBX stent	1 (20)
Operative time, minutes	61.8 (42-115)

*CTA,* Computed tomography angiography; *SBP,* systolic blood pressure.

At operative exploration, one patient was noted to have a return of arterial pulses consistent with vasospasm. The angiographic findings supported this clinical diagnosis. Thus, of the six surviving patients, a total of five stents were placed in five patients. Percutaneous femoral artery access was obtained in four of the five patients, and one patient had undergone brachial artery cutdown. Of the five stents used, four were self-expanding Gore Viabahn stents and one was a balloon-expandable Gore Viabahn VBX stent (Gore Medical, Flagstaff, Ariz). The VBX stent was placed in the innominate artery. Technical success was 100%. The mean duration of the procedure was 61.8 minutes (range, 42-105 minutes). No patient had required conversion to open repair. The mean length of stay for the patients with stents placed was 13.8 days (range, 2-29 days).

### Early (<30 days) complications

At 2 weeks after the initial intervention, patient 4 had presented with upper extremity numbness. Computed tomography angiography revealed an occluded subclavian stent (7-mm × 5-cm Viabahn; Gore Medical) despite dual antiplatelet therapy. The initial pre- and intraoperative images are shown in the Figure. An intravenous heparin infusion was started, and the patient underwent catheter-directed thrombolysis and thrombectomy with stent relining (6-mm × 6-cm Viabahn; Gore Medical) and was discharged with direct oral anticoagulant therapy.

**Fig:**
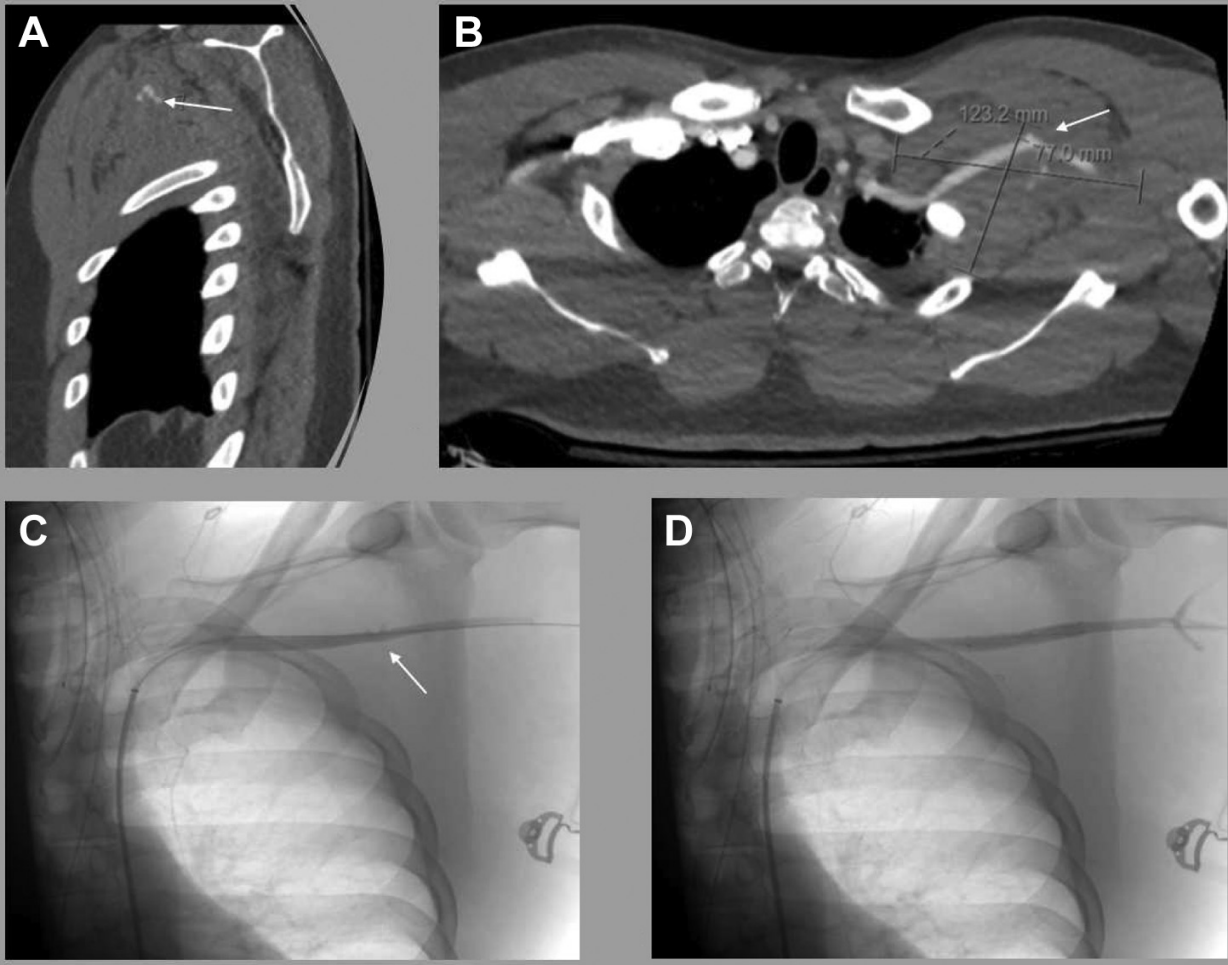
Preoperative and completion imaging findings for patient 4 with left subclavian artery transection. **A, B,** Computed tomography angiograms demonstrating 7.7 × 12.3-cm retroclavicular hematoma with areas of active arterial extravasation (*arrows*) within the hematoma. **C,** Intraoperative digital subtraction angiogram with femoral sheath access. Note arterial blush of the left subclavian artery (*arrow*). **D,** Completion digital subtraction angiogram after stent placement with no evidence of arterial extravasation.

### Long-term follow-up

A summary of the cohort's follow-up data is shown in Table III, with individual patient data presented in Table IV. The mean follow-up period was 48.6 weeks (range, 6-124 weeks), with three patients seen via telemedicine. All patients were asymptomatic, with no deaths.

**Table III:** Follow-up data

Variable	No. (%) or mean (range)
Stent thrombosis	1 (20)
Antiplatelet/anticoagulation compliance	2 (40)
Follow-up, weeks	48.6 (6-124)
Procedure-related amputation	0 (0)
Procedure-related mortality	0 (0)
Subsequent open revascularization	0 (0)
Length of stay, days	13.8 (2-29)

**Table IV:** Patient characteristics and follow up of patients treated with stent

Pt. No.	Sex; age, years	Injury mechanism	Injury location	Injury	SBP <90 mm Hg on arrival	Definitive signs of injury	Operative time, minutes	Early (<30 days) complications	Follow-up period, weeks	Follow-up findings (>30 days)
1	Male; 34	GSW	Left axillary	Vasospasm	No	No	115	None	Telephonic, 124	Asymptomatic; aspirin therapy
2	Male; 38	Stab	Left subclavian	Transection	Yes	Active pulsatile bleeding; pulses absent	52	Occlusion at 14 days requiring thrombectomy and relining	Clinic, 8	Patent stent on duplex ultrasound
3	Male; 26	GSW	Right axillary	Transection	Yes	Pulses absent	47	None	Telephonic,52	Asymptomatic
4	Male; 21	Blunt	Left subclavian	Occlusion	No	Pulses absent	42	None	Telephonic, 51	Patent stent on CTA at 4 weeks; asymptomatic at 51 weeks; aspirin therapy
5	Male; 21	GSW	Right innominate	PSA	No	Pulses absent	53	none	Clinic, 8	Asymptomatic; pulses intact

*CTA,* Computed tomography angiography; *GSW,* gunshot wound; *PSA,* pseudoaneurysm; *Pt. No.,* patient number; *SBP,* systolic blood pressure.

## Discussion

Despite improvements in operative techniques and treatment options, the mortality rate of traumatic SAVIs has remained as high as 30%.^3^^,^^11^^,^^14^ The high mortality has resulted partly from the difficulty of vessel exposure, which is complicated by the various structures in proximity to the thoracic outlet. The optimal treatment has remained unclear. The traditional reference standard has been an open approach, which requires a clavicular incision and an associated sternotomy or thoracotomy for as many as 50% of patients.^10^ Because of the associated morbidity, a shift has occurred toward endovascular-based therapy during the past two decades.^15^ Although the literature has remained limited to case series and small retrospective studies, the technical success rates for endovascular procedures have ranged from 66% to 100%, with most studies reporting no periprocedural mortality.^11^^,^^13^^,^^15^, ^16^, ^17^, ^18^, ^19^, ^20^

With the addition of endovascular intervention to treat these injuries, we are increasing our armamentarium. The proper selection of trauma patients suitable for endovascular repair is essential. Although most studies have included hemodynamically stable patients,^3^^,^^20^ we believe that endovascular intervention can be used as a stabilizing intervention in hemodynamically unstable patients, if not as definitive treatment, as shown in our study, albeit for a very limited sample. The use of an endovascular approach will help to overcome the issue of time-consuming dissection and the risk of collateral injury to the surrounding neurovascular structures.

The Endovascular Skills for Trauma and Resuscitative Surgery Working Group reported that endovascular procedures were successful in 96.9% of patients.^15^ Additionally, Branco et al^10^ evaluated 153 patients (18 endovascular and 135 open) after controlling for the injury severity score, blood pressure, Glasgow coma scale, and other demographic factors. They found significantly lower in-hospital mortality among the endovascular group (5.6% vs 27.8%; *P* = .04), in addition to a lower incidence of surgical site infections and sepsis.^10^

Ultimately, the long-term patency of covered stents in a historically young trauma patient population is of concern. The rate of graft thrombosis has been cited at 6% to 31% in the literature. However, the inconsistent follow-up has made these data inexact.^3^^,^^10^^,^^13^^,^^15^^,^^20^^,^^21^ Stents in this location are theoretically at an increased risk of occlusion owing to the compression and elongation of the stent between the first rib and clavicle in the thoracic outlet. In our cohort of five patients, one patient had developed early thrombosis despite antiplatelet therapy adherence. The patient's subclavian stent had likely been oversized as a 7-mm stent initially, because the stent was relined with a 6-mm stent after thrombolysis, which was patent on computed tomography angiography at 4 weeks. Although not studied in this particular location, excessive oversizing of self-expanding stents in the iliofemoral system has been associated with restenosis.^22^

Regarding surveillance, the rate of 1-year follow-up within trauma patients at our institution has been <20%. We have, thus, used trauma outreach coordinators to assess for effort-induced arm fatigue when telephone follow-up visits are possible. If a patient were symptomatic, immediate instructions to present to the hospital were provided.

Overall, we successfully used endovascular interventions to treat SAVIs. Endovascular repair can help stabilize a patient with possible concomitant injuries or provide time for adequate resuscitation. We have demonstrated that proper adoption of endovascular treatment of such morbid injuries can lead to a durable repair and leaves open the option for surgical bypass in the case of stent occlusion. The immediate complication rates were low; however, long-term management is essential.

## Conclusions

The results from our study have shown that endovascular treatment of trauma-related SAVIs can be performed safely and effectively with reduced operative times and morbidity compared with open exposure, although complications such as stent thrombosis can occur. Long-term data and prospective trials are needed to further investigate ideal patient selection and the outcomes of stenting vs open repair for this population.
